# Paroquet Springs

**Published:** 1840

**Authors:** 


### PAROQUET SPRINGS.

These Springs, which are beginning to attract much attention, having recently been improved in the most tasteful style, are situated on Salt river, in Bullitt county, within about twenty miles of Louisville. The valley in which they rise appears, from the shells which are found embedded in the earth, to have been the basin of an ancient lake, which has emptied its waters through Salt river into the Ohio, and thus found an outlet to the gulf. The geological formation is limestone. The valley is beautifully undulating, and is surrounded at the distance of a few miles by rugged hills.

The chief fountain belongs to the class of *Salino-Sulphur* Springs. It has been long in use, and is regarded by the people of the neighboring village of Shepherdsville, as highly medicinal. Since the property was purchased and improved by Mr. *Colmesnil*, the water of this Spring has been used by persons afflicted with rheumatism, liver and dyspeptic complaints, with great advantage. Cures of much interest have been reported to us by individuals in whose accuracy we can confide, and some we have ourselves seen. Among the rest, that of a lady, now resident at the Springs, is striking. She was supposed by her physicians to labor under an affection of the spinal cord, by which she was nearly deprived of the use of her arms, all motion of the shoulders being painful and difficult. At the time that she removed to the Springs and commenced the use of the water, she was comparatively helpless; unable to bear the slighest exposure to cold, and incapable of any but the gentlest exercise. Her digestion was also bad, and she was troubled especially with cardialgia and constipated bowels. This was in December. She has used the water internally, and by sponging, and as a bath, and the benefit derived from it is most manifest. In a few weeks, she was relieved of the pain and stiffness of the shoulders, and was able to walk over the grounds, even in the winter season, with impunity. Her digestion is restored, and she considers her health good.

On analysis, this Spring has been found to contain the following ingredients:

1. Carbonic acid gas, (copious:)
2. Sulphuretted hydrogen gas:
3. Muriate of soda, (abundant:)
4. Muriate of lime:
5. Muriate of magnesia:
6. Sulphate of soda:
7. Sulphates of magnesia and lime:
8. Carbonates of lime, magnesia, and probably soda.

Its peculiar taste and odor are imparted by the sulphuretted hydrogen and muriate of soda, with which it is largely impregnated; and the quantity of muriate of lime and muriate of magnesia in it, is also sufficient to affect its medicinal virtues. Three tumblers' full of the water will move the bowels of most persons, and powerfullly excite the kidneys. Cutaneons eruptions not unfrequently appear after it has been in use for a few days, but these give place to a very healthy state of the skin. In composition, it is nearly identical with the famous Harrowgate and Dinsdale Springs in England, and with the Blue Lick, and Olympian Springs of this State, long places of resort for invalids, and is, of course, adapted to that class of affections in which they have been found useful.

A second spring issues within a few paces of the one just described, of the same chemical constitution, so far as we could ascertain, but which is said to possess the singular quality of exciting nausea and free vomiting, even when drunk in moderate quantities. We have not had an opportunity of testing its virtues in this way. It is not so highly charged with the muriate of soda or sulphuretted hydrogen, and contains a greater proportion of the muriates of lime and magnesia than its neighbor, and this is the only difference we have been able to detect. It may, however, give up on future trial some element which, hitherto, has eluded our analysis.

The third is called the ***Epsom Spring***, and issues from the brink of a hill three hundred yards distant from the others. Its taste is bitter, resembling that of Epsom salts, the presence of which substance gives to it its peculiar properties. To most persons it is less palatable than the salt-sulphur water, and, consequently, its remedial qualities have not been so fully tested. It acts decidedly upon the bowels, and also promotes the action of the kidneys.

The following is the result of our analysis of this water:

1. Carbonic acid gas:
2. Sulphate of Magnesia, (abundant:)
3. Sulphate of lime:
4. Carbonate of lime:
5. Carbonate of Magnesia:
6. Muriate of lime:
7. Muriate of Magnesia:

This spring bears a close analogy to the Epsom water of England, and one of the springs at Harrodsburg, the medical virtues of which have been confirmed by the experience of many years. Invalids might find it beneficial to alternate the use of it with that of the Salino-Sulphur Spring. Of the efficacy of the latter in dyspeptic complaints there cannot be a doubt, and it may, therefore, be safely recommended to that numerous class of sufferers. Y.

